# The effect of different combinations of flaxseed, melatonin, gum acacia, and betaine on diabetic rats with adenine-induced chronic kidney disease

**DOI:** 10.3389/fphar.2025.1600484

**Published:** 2025-09-10

**Authors:** Mohammed Al Za’abi, Yousuf Al Suleimani, Haytham Ali, Badreldin H. Ali, Raya Al Maskari

**Affiliations:** ^1^ Department of Pharmacology and Clinical Pharmacy, College of Medicine and Health Sciences, Sultan Qaboos University, Al Khod, Oman; ^2^ Department of Animal and Veterinary Sciences, College of Agriculture and Marine Sciences, Sultan Qaboos University, Al Khod, Oman

**Keywords:** diabetes, chronic kidney disease, gum acacia, melatonin, betaine, flaxseed

## Abstract

Diabetes mellitus (DM) and chronic kidney disease (CKD) are associated with significant morbidity and mortality. Their progression is driven by inflammation, oxidative stress, and apoptosis. In this study, we examined the effects of nine different combinations of gum acacia (GA), melatonin, betaine, and flaxseed—used in pairs or trios—on adenine-induced CKD in streptozotocin (STZ)-induced diabetic rats. Rats treated with adenine and STZ exhibited significant hyperglycemia and CKD manifestations such as elevated plasma levels of cystatin C and indoxyl sulfate, increased urinary levels of N-acetyl-β-D-glucosaminidase (NAG) and NAG/creatinine ratio, and reduced creatinine clearance. Additionally, there was a significant decrease in renalase activity and urine osmolality, alongside a significant increase in IL-1β, IL-6, and TNF-α levels and a decrease in IL-10 levels. Oxidative stress biomarkers including superoxide dismutase, glutathione reductase, total antioxidant capacity, and catalase activities were also significantly impaired. These findings were supported by histopathological changes consistent with CKD. Treatment with the combinations of two or three agents alleviated most of these changes to varying degrees. Notably, the GA–melatonin–betaine combination demonstrated the most significant improvement across all the parameters along with the preservation of the kidney tissue structure. These improvements may partially be explained by the enhanced glycemic control achieved by this combination, in addition to possible synergistic molecular, pharmacokinetic, and pharmacodynamic interactions. These findings support the potential of this combination to attenuate the progression of CKD in the setting of diabetes. However, further mechanistic studies, pharmacokinetic profiling, and long-term toxicity data are necessary to validate its efficacy and safety for clinical use.

## 1 Introduction

Diabetes mellitus (DM) is a major global health problem, affecting over 537 million adults aged 20–79 years worldwide (Sun et al., 2022). It is associated with numerous micro- and macro-vascular complications, including nephropathy, neuropathy, and retinopathy, all of which significantly increase morbidity, mortality, and health-care costs. Diabetic nephropathy results from glomerular capillary damage and may progress to chronic kidney disease (CKD), ultimately requiring dialysis or kidney transplantation (Arora and Singh, 2013). CKD, regardless of the etiology, contributes significantly to the global disease burden and affects more than 200 million individuals worldwide. Each year, millions die from CKD due to limited access to treatment (Jha and Modi, 2018).

Inflammation, oxidative stress, and apoptosis play central roles in the pathogenesis and progression of both DM and CKD (Akchurin and Kaskel, 2015; Tsalamandris et al., 2019). Patients with DM and/or CKD often exhibit low-grade but persistent inflammation, which contributes to disease complications and increased mortality (Duncan et al., 2003). Elevated levels of inflammatory and oxidative biomarkers are inversely associated with renal function and have been linked to the micro- and macro-vascular complications associated with diabetes (Qian, 2017). In experimental models of CKD, key inflammatory mediators—including C-reactive protein, tumor necrosis factor, and various cytokines—and markers of oxidative and nitrosative stress exert similar pathogenic effects (Kurose et al., 2013). These findings support the potential role of anti-inflammatory and antioxidant agents as adjunctive therapies for patients with DM and CKD.

Currently, there is no cure for DM; therefore, pharmacological management aims to either enhance endogenous insulin secretion (e.g., sulphonylureas) or improve insulin sensitivity (e.g., thiazolidinediones and the biguanide metformin). The chronic nature of DM, coupled with the often-delayed onset of complications, may lead patients to underestimate the severity of their condition. This often contributes to poor adherence to prescribed therapies and an increased tendency to explore alternative treatments, including herbal medicine (Cefalu et al., 2011; Forbes and Cooper, 2013).

Despite global advancements, plant-based medicines continue to be widely used for managing various pathological conditions, either as substitutes or as adjuncts for conventional medications. Many patients incorporate herbal extracts into their regimens based on the perception that they are “natural” and “do no harm,” even in the absence of robust evidence regarding their effectiveness or safety (Cefalu et al., 2011; Rutebemberwa et al., 2013). This underscores the need for rigorously designed laboratory, animal, and clinical studies to elucidate the mechanism of action of these agents and to establish their efficacy and, more importantly, their safety. Several compounds such as flaxseed, gum acacia (GA), melatonin, and betaine have demonstrated potential in preclinical and clinical studies involving patients with CKD and/or diabetes.

Flaxseed (*Linum usitatissimum*) has been traditionally used in folk medicine for the treatment of various ailments (Mueed et al., 2022). It contains 32%–45% oil, of which 51%–55% is α-linolenic acid. In addition, flaxseed is a rich source of phenolic compounds including phenolic acids, flavonoids, and lignans. Among these, secoisolariciresinol diglucoside has been shown to exhibit antioxidant, hypolipidemic, and hypoglycemic properties (Mueed et al., 2022). Preclinical studies have shown that flaxseed may attenuate the progression of experimentally induced CKD in diabetic rat models (Al Za’abi et al., 2021b), supporting its potential as a therapeutic agent in DM and CKD.

Melatonin, a hormone produced by the pineal gland, functions as an antioxidant and has been shown to support immune function, scavenge free radicals, and exert cardioprotective and neuroprotective effects (Samanta, 2022). It has also been demonstrated to mitigate cisplatin-induced nephrotoxicity, both as monotherapy and in combination with other agents (Ali et al., 2020).

Betaine (trimethylglycine) is a naturally occurring amino acid present in a variety of microorganisms, plants, and animals, including seafood, wheat germ, and spinach (Dobrijević et al., 2023). It plays a key role in methylation reactions and contributes to homocysteine reduction (McRae, 2013). In addition to its antioxidant and anti-inflammatory properties, betaine has demonstrated therapeutic potential in conditions such as obesity, cancer, and Alzheimer’s disease (Zhao et al., 2018). Furthermore, it has been shown to enhance renal function and protect kidneys against tetrachloride-induced nephrotoxicity (Ozturk et al., 2003). Notably, the combination of betaine and melatonin has been reported to provide greater protection against cisplatin-induced nephrotoxicity than either agent alone (Al Za’abi et al., 2021a).

GA is a prebiotic, water-soluble dietary fiber and complex heteropolysaccharide derived from *Acacia senegal* or *Acacia seyal* trees. Multiple experimental and clinical studies have demonstrated the beneficial effects of GA in CKD (Ali et al., 2010; 2013a). In adenine-induced CKD models, GA has been shown to improve various biochemical, physiological, and behavioral parameters (Ali et al., 2015).

Recent evidence suggests that combining nephroprotective agents may offer enhanced efficacy compared to monotherapy (Casanova et al., 2020). Therefore, in this study, we aimed to examine the efficacy and/or potential toxicity of different combinations of GA, melatonin, betaine, and flaxseed in a rat model of adenine-induced CKD with experimentally induced diabetes.

## 2 Materials and methods

### 2.1 Animals

Male Wistar rats were obtained from the small animal house, Sultan Qaboos University (SQU), Oman, and housed in a room with a regulated environment (temperature 22 °C ± 2 °C, relative humidity approximately 60%, and 12 h light–dark cycle, with light at 6.00 a.m.), and they were fed a standard additive-free diet and tap water *ad libitum.* The use of rats was approved by the SQU Ethical Committee for Animal Use in Research (SQU/EC-AUR/2023–2024/2). All procedures involving the animals and their care were carried out in accordance with the guidelines of the national and international laws and policies.

### 2.2 Experimental design

A sample size of six rats per group was chosen based on the standard practices in preclinical studies and published guidelines (Charan and Kantharia, 2013). Rats (n = 66) were randomly assigned into 11 equal groups and treated as follows:

• G1 (control): received distilled water.
• G2 (adenine + streptozotocin): received an intraperitoneal dose of 50 mg/kg of streptozotocin, dissolved in 0.1 M citrate buffer (pH 4.5), to induce DM and then adenine 0.25% w/w in the feed for 4 weeks to induce CKD.
• G3 (M + B): similar to G2 and additionally received melatonin (suspended in 0.9% saline at a dose of 10 mg) and betaine (suspended in 0.9% saline at an oral dose of 200 mg/kg/day).
• G4 (G + B): similar to G2 and additionally received GA in drinking water at a concentration of 15% w/v and betaine (suspended in 0.9% saline at an oral dose of 200 mg/kg/day).
• G5 (F + B): similar to G2 and additionally received flaxseed (15% w/w) and betaine (suspended in 0.9% saline at an oral dose of 200 mg/kg/day).
• G6 (M + G): similar to G2 and additionally received melatonin (suspended in 0.9% saline at an oral dose of 10 mg) and GA in drinking water at a concentration of 15% w/v.
• G7 (M + F): similar to G2 and additionally received melatonin (suspended in 0.9% saline at an oral dose of 10 mg) and flaxseed (15% w/w).
• G8 (F + G): similar to G2 and additionally received flaxseed (15% w/w) and GA in drinking water at a concentration of 15% w/v.
• G9 (F + G + M): similar to G2 and additionally received flaxseed (15% w/w), GA in drinking water at a concentration of 15% w/v, and melatonin (suspended in 0.9% saline at an oral dose of 10 mg).
• G10 (F + M + B): similar to G2 and additionally received flaxseed (15% w/w), melatonin (suspended in 0.9% saline at an oral dose of 10 mg), and betaine (suspended in 0.9% saline at a dose of 200 mg/kg/day).
• G11 (G + M + B): similar to G2 and additionally received GA in drinking water at a concentration of 15% w/v, melatonin (suspended in 0.9% saline at an oral dose of 10 mg), and betaine (suspended in 0.9% saline at an oral dose of 200 mg/kg/day).

The doses were selected based on previous studies from our group and others, which demonstrated their efficacy and safety in similar animal models (Al Za’abi et al., 2018; Al Za’abi et al., 2021a; Al Za’abi et al., 2021b).

One day before the end of the treatment period, rats were individually placed in metabolic cages, and urine was collected over a 24-h period. At the end of the treatment period, rats were anesthetized via intraperitoneal injection of ketamine (75 mg/kg) and xylazine (5 mg/kg). Blood was collected from the abdominal aorta in heparinized tubes, and the plasma was harvested by centrifugation at 900 g at 4 °C for 15 min. The rats were then euthanized by anesthetic overdose, and the two kidneys were excised. A small section of the right kidney was fixed in 10% formalin for histological analysis. The remainder of the right kidney and the left kidney were dipped in liquid nitrogen and frozen at −80 °C for further analysis.

### 2.3 Drugs and chemicals

Streptozotocin, betaine, adenine, and GA were purchased from Sigma-Aldrich (Saint Louis, MO, United States). Melatonin was purchased from Glentham Life Sciences (Unit 5 Leafield Way, Corsham, UK). Flaxseed was purchased from Badia Spices Inc. (Doral, FL, United States). The plasma urea, creatinine, calcium, phosphorous, uric acid, and urine albumin were measured using the fully automated chemistry analyzer BS-120, Mindray (Shenzhen, CHINA). Urine N-acetyl-β-D-glucosaminidase (NAG), plasma neutrophil gelatinase-associated lipocalin (NGAL), cystatin C, interleukin-6 (IL-6), interleukin-1 beta (IL-1β), interleukin-10 (IL-10), 8-hydroxy-2′-deoxyguanosine (8-OHdG), and tumor necrosis factor alpha (TNF-α) were measured using ELISA kits from Elabscience Bionovation Inc. (Houston, Texas, United States). Plasma 8-isoprostane, advanced glycation end products (AGEs), indoxyl sulfate, and renalase were measured using ELISA kits from Assay Genie Ltd. (Windsor Place, Dublin, Ireland). Glutathione reductase (GR) was measured using colorimetric assay kits from Bio-vision (Milpitas, CA, United States). superoxide dismutase (SOD), total antioxidant capacity (TAC), and catalase were measured using colorimetric assay kits from Elabscience Bionovation Inc. (Houston, Texas, United States). Urine osmolality was measured using a freezing point osmometer (Gonotec, GmbH, Berlin, Germany).

### 2.4 Histological analysis

Paraffin-embedded renal tissue sections were stained with hematoxylin and eosin (H&E) and Picrosirius red (ab150681, Abcam). Renal tubular necrosis was assessed following the study by Ali et al. (2013b) using a scoring method on a scale of 0–4, where 0 = normal, no necrosis; 1 < 10%; 2 = 10%–25%; 3 = 26%–75%; 4 > 75%. Three 40X microscopic fields were analyzed from each kidney section of each animal of the 11 groups, and the score was calculated according to the mean percentage. Fibrosis was assessed using the Picrosirius red stain.

### 2.5 Statistical analysis

Data are given as the mean ± SEM and were analyzed by one-way analysis of variance followed by Bonferroni’s multiple comparison tests (GraphPad Prism version 5.03, San Diego, CA, United States).

## 3 Results

### 3.1 Physiological parameters

The effects of melatonin, betaine, GA, and flaxseed on the physiological parameters in rats with diabetes and adenine-induced CKD are summarized in Table 1. As shown in the table, the initial body weights were comparable across the groups. Treatment with adenine and STZ caused a significant reduction in body weight change and a significant increase in relative kidney weights, liver weights, water intake, urine flow, feed intake, and fecal output compared with the control group. Treatment with the different combinations significantly ameliorated the induced changes in relative kidney weight and urine flow. The flaxseed–betaine, flaxseed–melatonin, flaxseed–GA, flaxseed–melatonin–betaine, and GA–melatonin–betaine combinations significantly ameliorated the induced changes in body weight. The GA–betaine, GA–melatonin, flaxseed–melatonin, flaxseed–GA, flaxseed–melatonin–betaine, and flaxseed–melatonin–GA combinations significantly ameliorated the induced changes in liver weight. Adenine- and STZ-induced changes in feed intake were significantly reversed by the melatonin–GA, melatonin–flaxseed, GA–flaxseed–melatonin, and GA–melatonin–betaine combinations. All the combinations except GA–melatonin–betaine significantly ameliorated the induced changes in the fecal output.

**TABLE 1:** Effect of treatment with melatonin(M), betaine(B), gum acacia (G), and flaxseed (F) on some physiological parameters in rats with both diabetes (D) and adenine (A)-induced chronic kidney disease (CKD).

Parameters/treatments	Group 1 Control	Group 2 A + D	Group 3 A + D + M + B	Group 4 A + D + G + B	Group 5 A + D + F + B	Group 6 A + D + M + G	Group 7 A + D + M + F	Group 8 A + D + F + G	Group 9 A + D + F + G + M	Group 10 A + D + F + M + B	Group 11 A + D + G + M + B
Initial body weight(g)	273.2 ± 14.89	274.3 ± 15.5	275.2 ± 6.59	274.0 ± 9.92	274.0 ± 17.06	275.3 ± 7.21	269.2 ± 7.59	276.2 ± 12.81	274.2 ± 2.02	274.5 ± 2.29	271.3 ± 3.56
Final body weight (g)	334.2 ± 11.68	198.3 ± 15.29^a^	215.0 ± 12.58 ^a^	208.0 ± 9.4^a^	260.7 ± 12.4^a^ ^b^ ^c^ ^d^	234.0 ± 10.82 ^a^	251.0 ± 9.27 ^a^ ^b^	266.8 ± 10.87 ^a^ ^b^ ^c^ ^d^	202.5 ± 4.43^a^ ^e^ ^g^ ^h^	242.5 ± 14.28 ^a^ ^i^	270.7 ± 4.78 ^a^ ^b^ ^c^ ^d^
Body weight change (%)	23.7 ± 6.76	−27.7 ± 3.92^a^	−22.2 ± 2.72^a^	−24.2 ± 1.4^a^	−4.3 ± 2.32^a^ ^b^ ^c^ ^d^	−15.2 ± 2.14^a^	−6.8 ± 0.99 ^a^ ^b^ ^c^ ^d^	−3.2 ± 1.8 ^a^ ^b^ ^c^ ^d^	−26.1 ± 1.97 ^a^ ^g^ ^h^	−11.6 ± 5.4 ^a^ ^b^ ^e^ ^i^	−0.27 ± 0.56 ^a^ ^b c d f j^
Relative kidney weight (%)	0.54 ± 0.01	1.01 ± 0.03 ^a^	0.84 ± 0.08 ^a^ ^b^	0.77 ± 0.02 ^a^ ^b^	0.8 ± 0.04 ^a^ ^b^	0.76 ± 0.04 ^a^ ^b^	0.71 ± 0.02 ^a^ ^b^	0.7 ± 0.03 ^a^ ^b^	0.67 ± 0.02 ^b^ ^c^	0.73 ± 0.02 ^a^ ^b^	0.74 ± 0.04 ^a^ ^b^
Relative liver weight (%)	2.79 ± 0.05	4.2 ± 0.11 ^a^	3.82 ± 0.29 ^a^	3.5 ± 0.09 ^a^ ^b^	3.74 ± 0.17 ^a^	3.43 ± 0.05 ^a^ ^b^	3.14 ± 0.06 ^b^ ^c^ ^e^	3.26 ± 0.16^b^	3.16 ± 0.07 ^b^ ^c^ ^e^	3.45 ± 0.11 ^a^ ^b^	3.72 ± 0.23 ^a^ ^g^ ^i^
Water intake (mL)	17.2 ± 1.49	143.3 ± 12.7 ^a^	99.7 ± 6.22 ^a^ ^b^	89.5 ± 6.17 ^a^ ^b^	93.3 ± 12.03 ^a^ ^b^	92.0 ± 2.74 ^a^ ^b^	80.8 ± 8.09 ^a^ ^b^	82.3 ± 6.8 ^a^ ^b^	58.8 ± 6.39 ^a^ ^b c d^ ^e^ ^f^	66.3 ± 6.03 ^a^ ^b^ ^c^	37.2 ± 3.56 ^b^ ^c^ ^d^ ^e^ ^f^ ^g^ ^h^
Urine flow (μL/min)	6.9 ± 0.95	74.4 ± 7.84^a^	40.6 ± 5.04 ^a^ ^b^	40.3 ± 4.82 ^a^ ^b^	49.7 ± 5.77 ^a^ ^b^	52.1 ± 1.45 ^a^ ^b^	47.0 ± 3.51 ^a^ ^b^	50.0 ± 5.05 ^a^ ^b^	30.3 ± 2.57 ^a^ ^b f g h^	44.2 ± 3.05 ^a^ ^b^ ^e^ ^i^	18.7 ± 0.51 ^b^ ^c^ ^d^ ^e^ ^f^ ^g^ ^h^ ^i^
Feed intake (g)	19.2 ± 1.31	27.73 ± 2.61^a^	19.00 ± 2.8	24.7 ± 1.57	28.67 ± 3.50 ^a^ ^c^	21.08 ± 1.95 ^b^	20.55 ± 0.51 ^b^	25.93 ± 2.03 ^a^	11.6 ± 0.66 ^a^ ^b^ ^d^ ^e^	19.0 ± 1.42 ^e^	14.5 ± 1.34 ^b^ ^d^ ^e^
Faecal output (g)	5.0 ± 0.81	15.4 ± 1.33^a^	7.08 ± 0.57^b^	14.0 ± 0.72^a^ ^c^	17.4 ± 2.84^a^ ^d^	9.3 ± 1.02 ^b^ ^e^	7.2 ± 0.53 ^b^ ^d^ ^e^	10.5 ± 1.28 ^a^ ^e^	7.3 ± 0.82 ^b^ ^d^ ^e^	9.5 ± 1.23 ^b^ ^d^ ^e^	7.5 ± 1.96 ^b^ ^d^ ^e^

Values in the table are means ± SEM (n = 6).

Diabetes (D) was induced by a single intraperitoneal injection of streptozotocin (55 mg/kg) on the first day of the experiment; after diabetes confirmation, CKD was induced by the inclusion of adenine (A) in the feed at a concentration of 0.25%^w/w^ for 35 days, and M (10 mg/kg) and B (200 mg/kg) were administered concurrently to rats by oral gavage. G (15%^w^/^v^) was added in drinking water. F (15%^w/w^) was administered in a powdered form in the food. On the 35th day of treatment, the rats were placed in metabolic cages for urine collection.

Differences between groups were assessed by one-way analysis of variance (ANOVA) followed by Bonferroni’s multiple comparison test. Statistical significance was set at *p* < 0.05. Superscripts indicate the following comparisons:

^a^ Control group vs. all other groups.

^b^ A + D (untreated) group vs. all other A + D treated groups.

^c^ A + D + M + B group vs. all other A + D treated groups.

^d^ A + D + G + B group vs. all other A + D treated groups.

^e^ A + D + F + B group vs. all other A + D treated groups.

^f^ A + D + M + G group vs. all other A + D treated groups.

^g^ A + D + M + F group vs. all other A + D treated groups.

^h^ A + D + F + G group vs. all other A + D treated groups.

^i^ A + D + F + G + M group vs. all other A + D treated groups.

^j^ A + D + F + M + B group vs. all other A + D treated groups.

### 3.2 Blood glucose levels

The effects of melatonin, betaine, GA, and flaxseed on various plasma renal parameters and blood glucose levels in rats with diabetes and adenine-induced CKD are summarized in Table 2. The adenine and STZ group had significantly higher fasting blood glucose levels (32.7 ± 0.30 mmol/L) than the control group (4.2 ± 0.11 mmol/L). All the treatment combinations except the GA–betaine combination significantly reduced these levels. The combination of GA–melatonin–betaine yielded the most substantial reduction (7.5 ± 0.48 mmol/L) compared with the other treatment combinations.

**TABLE 2:** Effect of treatment with melatonin (M), betaine (B), gum acacia (G), and flaxseed (F) on some plasma parameters in rats with both diabetes (D) and adenine (A)-induced chronic kidney disease (CKD).

Parameters/treatments	Group 1 Control	Group 2 A + D	Group 3 A + D + M + B	Group 4 A + D + G + B	Group 5 A + D + F + B	Group 6 A + D + M + G	Group 7 A + D + M + F	Group 8 A + D + F + G	Group 9 A + D + F + G + M	Group 10 A + D + F + M + B	Group 11 A + D + G + M + B
Fasting blood sugar (mmol/L)	4.2 ± 0.11	32.7 ± 0.30^a^	22.5 ± 10.81 ^a^ ^b^	23.9 ± 2.81 ^a^	16.2 ± 2.54^a^ ^b^	20.2 ± 3.40 ^a^ ^b^	13.6 ± 2.88^b^ ^d^	8.9 ± 1.73^b^ ^c^ ^d^ ^f^	21.5 ± 1.12 ^a^ ^b^ ^h^	15.0 ± 4.36 ^a^ ^b^	7.5 ± 0.48 ^b^ ^c^ ^d^ ^f^ ^i j^
Urea (mmol/L)	3.40 ± 0.43	11.84 ± 1.96^a^	8.79 ± 1.11^a^	10.75 ± 0.66^a^	8.19 ± 0.82^a^	6.99 ± 0.38^b^ ^d^	4.57 ± 0.46 ^b^ ^c^ ^d^ ^e^	6.68 ± 0.66 ^b^ ^d^	4.77 ± 0.55 ^b^ ^c^ ^d^ ^e^	6.77 ± 1.21^b^ ^d^	3.91 ± 0.28^b^ ^c^ ^d^ ^j^
Creatinine (µmol/L)	15.6 ± 1.49	52.1 ± 3.59^a^	43.1 ± 1.20^a^	41.7 ± 3.14^a^	35.2 ± 3.77^a^	33.4 ± 3.07^a^ ^b^ ^c^	32.0 ± 3.08 ^a^ ^b^ ^c^	28.8 ± 3.19 ^a^ ^b^ ^c^	29.9 ± 2.26^a^ ^b^ ^c^	31.6 ± 1.97 ^a^ ^b^ ^c^	21.4 ± 1.64 ^b^ ^c^ ^d^ ^e^ ^f^ ^g^ ^j^
Uric acid (µmol/L)	22.2 ± 0.41	58.4 ± 6.95^a^	36.7 ± 1.32^a^	32.8 ± 3.0^b^	26.5 ± 1.9^b^	32.7 ± 1.19^b^	27.8 ± 2.58 ^b^	32.6 ± 2.54 ^b^	30.2 ± 2.36 ^b^	29.8 ± 1.8^b^	22.6 ± 1.33 ^b^ ^c^ ^h^
Calcium (mmol/L)	0.76 ± 0.04	0.44 ± 0.03^a^	0.58 ± 0.07	0.68 ± 0.05^b^	0.63 ± 0.05^b^	0.69 ± 0.02 ^b^	0.58 ± 0.04 ^b^	0.68 ± 0.05^b^	0.64 ± 0.06 ^b^	0.68 ± 0.06^b^	0.70 ± 0.02^b^
Phosphorous (mmol/L)	0.69 ± 0.06	1.44 ± 0.18^a^	1.00 ± 0.09	1.32 ± 0.17 ^a^	1.25 ± 0.15^a^	1.03 ± 0.06	1.17 ± 0.07	1.03 ± 0.13	0.86 ± 0.2 ^b^	1.07 ± 0.18	0.82 ± 0.07 ^b^

Values in the table are means ± SEM (n = 6).

Diabetes (D) was induced by a single intraperitoneal injection of streptozotocin (55 mg/kg) on the first day of the experiment; after diabetes confirmation, CKD was induced by the inclusion of adenine (A) in the feed at a concentration of 0.25%^w/w^ for 35 days, and M (10 mg/kg) and B (200 mg/kg) were administered concurrently to rats by oral gavage. G (15%^w/v^) was added in drinking water. F (15%^w/w^) was administered in a powdered form in the food. On the 35th day of treatment, the rats were placed in metabolic cages for urine collection.

Differences between the groups were assessed by one-way analysis of variance (ANOVA) followed by Bonferroni’s multiple comparison test. Statistical significance was set at *p* < 0.05. Superscripts indicate the following comparisons:

^a^ control group vs. all other groups.

^b^ A + D (untreated) group vs. all other A + D treated groups.

^c^ A + D + M + B group vs. all other A + D treated groups.

^d^ A + D + G + B group vs. all other A + D treated groups.

^e^ A + D + F + B group vs. all other A + D treated groups.

^f^ A + D + M + G group vs. all other A + D treated groups.

^g^ A + D + M + F group vs. all other A + D treated groups.

^h^ A + D + F + G group vs. all other A + D treated groups.

^i^ A + D + F + G + M group vs. all other A + D treated groups.

^j^ A + D + F + M + B group vs. all other A + D treated groups.

### 3.3 Biochemical and urinary parameters

Adenine and STZ caused a significant decrease in plasma calcium levels and significantly increased phosphorus, uric acid, urea, and creatinine plasma levels (Table 2). All the treatment combinations significantly mitigated the changes in calcium and uric acid levels. On the other hand, only the flaxseed–GA–melatonin and GA–melatonin–betaine combinations significantly attenuated the changes in phosphorus levels. All the treatment combinations, except melatonin–betaine, GA–betaine, and flaxseed–betaine, attenuated the changes in urea and creatinine plasma levels. The melatonin–flaxseed, flaxseed–GA–melatonin, and GA–melatonin–betaine combinations yielded the most pronounced and significant reductions in urea levels, whereas the GA–melatonin–betaine combination yielded the most notable and significant reduction in creatinine levels compared to the other treatment combinations.

Adenine and STZ induced significant changes in the urinary creatinine levels, NAG levels, albumin/creatinine ratios, creatinine clearance, NAG/creatinine ratios, and urine osmolality compared with the control group (Table 3). The GA–melatonin–betaine combination improved all these changes, except the albumin/creatinine ratios. Treatment with other different combinations, except GA–betaine, significantly improved the NAG/creatinine ratios and osmolality.

**TABLE 3:** Effect of treatment with melatonin (M), betaine (B), gum acacia (G), and flaxseed (F) on some urine parameters in rats with both diabetes (D) and adenine (A)-induced chronic kidney disease (CKD).

Parameters/treatments	Group 1 Control	Group 2 A + D	Group 3 A + D + M + B	Group 4 A + D + G + B	Group 5 A + D + F + B	Group 6 A + D + M + G	Group 7 A + D + M + F	Group 8 A + D + F + G	Group 9 A + D + F + G + M	Group 10 A + D + F + M + B	Group 11 A + D + G + M + B
Creatinine (µmol/L)	8514.0 ± 775.8	364.7 ± 99.1^a^	619.5 ± 96.4^a^	417.1 ± 46.8^a^	952.8 ± 434.9^a^	600.5 ± 95.6^a^	1045.1 ± 226.0^a^	1097.0 ± 462.2^a^	871.3 ± 66.2^a^	871.6 ± 92.4^a^	3493.9 ± 180.7 ^a^ ^b^ ^c^ ^d^ ^f^ ^g^ ^h^ ^i^
NAG (ng/mL)	3.71 ± 0.40	14.47 ± 1.30^a^	9.39 ± 1.21^a^ ^b^	9.10 ± 0.47^a^ ^b^	6.68 ± 0.52^b^	6.37 ± 0.92^b^	6.44 ± 1.23^b^	4.83 ± 0.49^b^ ^c^ ^d^	4.91 ± 0.42^b^ ^c^ ^d^	5.05 ± 0.41^b^ ^c^ ^d^	4.37 ± 0.35^b^ ^c^ ^d^
Albumin/creatinine ratio (mg/μmol)	0.54 ± 0.08	2.43 ± 0.53^a^	2.24 ± 0.23^a^	1.25 ± 0.15	2.32 ± 0.63^a^	0.92 ± 0.10^b^ ^c^ ^e^	1.13 ± 0.25 ^b^ ^e^	1.66 ± 0.30	1.37 ± 0.13	1.22 ± 0.32	1.22 ± 0.08
Creatinine clearance (mL/minute)	3.74 ± 0.41	0.49 ± 0.13^a^	0.61 ± 0.17^a^	0.41 ± 0.07^a^	1.31 ± 0.55^a^	1.04 ± 0.25^a^	1.57 ± 0.35^a^	1.88 ± 0.70^a^	0.89 ± 0.08^b^ ^c^	1.23 ± 0.17^a^	3.13 ± 0.28^b^ ^c^ ^d^ ^f^ ^g^ ^h^ ^i^
NAG/creatinine ratio (ng/µmol)	0.47 ± 0.09	57.37 ± 15.75^a^	17.52 ± 3.59^b^	23.99 ± 3.98^a^	12.84 ± 3.12^b^	13.83 ± 4.21^b^	8.16 ± 2.82^b^	7.27 ± 2.20^b^	5.86 ± 0.78 ^b^	6.18 ± 0.90 ^b^	1.27 ± 0.13^b^ ^d^
Osmolality (mOsmol/kg)	1710.2 ± 142.7	656.0 ± 37.7^a^	810.0 ± 33.6^a^	759.2 ± 56.4^a^	981.0 ± 60.8^a^	947.8 ± 45.2 ^a^	1074.3 ± 111.7^a^ ^b^	1179.8 ± 175.3^a^ ^b^ ^d^	1052.3 ± 46.2^a^ ^b^	1165.3 ± 42.7^a^ ^b^ ^d^	1726.2 ± 127.0 ^b^ ^c^ ^d^ ^f^ ^g^ ^h^ ^i^ ^j^

Values in the table are means ± SEM (n = 6).

Diabetes (D) was induced by a single intraperitoneal injection of streptozotocin (55 mg/kg) on the first day of the experiment; after diabetes confirmation, CKD was induced by the inclusion of adenine (A) in the feed at a concentration of 0.25%^w/w^ for 35 days, and M (10 mg/kg) and B (200 mg/kg) were administered concurrently to rats by oral gavage. G (15%^w/v^) was added in drinking water. F (15%^w/w^) was administered in a powdered form in the food. On the 35th day of treatment, the rats were placed in metabolic cages for urine collection. NAG: N-acetyl-β-D-glucosaminidase.

Differences between groups were assessed by one-way analysis of variance (ANOVA) followed by Bonferroni’s multiple comparison test. Statistical significance was set at p < 0.05. Superscripts indicate the following comparisons:

^a^ control group vs. all other groups.

^b^ A + D (untreated) group vs. all other A + D treated groups.

^c^ A + D + M + B group vs. all other A + D treated groups.

^d^ A + D + G + B group vs. all other A + D treated groups.

^e^ A + D + F + B group vs. all other A + D treated groups.

^f^ A + D + M + G group vs. all other A + D treated groups.

^g^ A + D + M + F group vs. all other A + D treated groups.

^h^ A + D + F + G group vs. all other A + D treated groups.

^i^ A + D + F + G + M group vs. all other A + D treated groups.

^j^ A + D + F + M + B group vs. all other A + D treated groups.

### 3.4 Renal plasma indices

**FIGURE 1:**
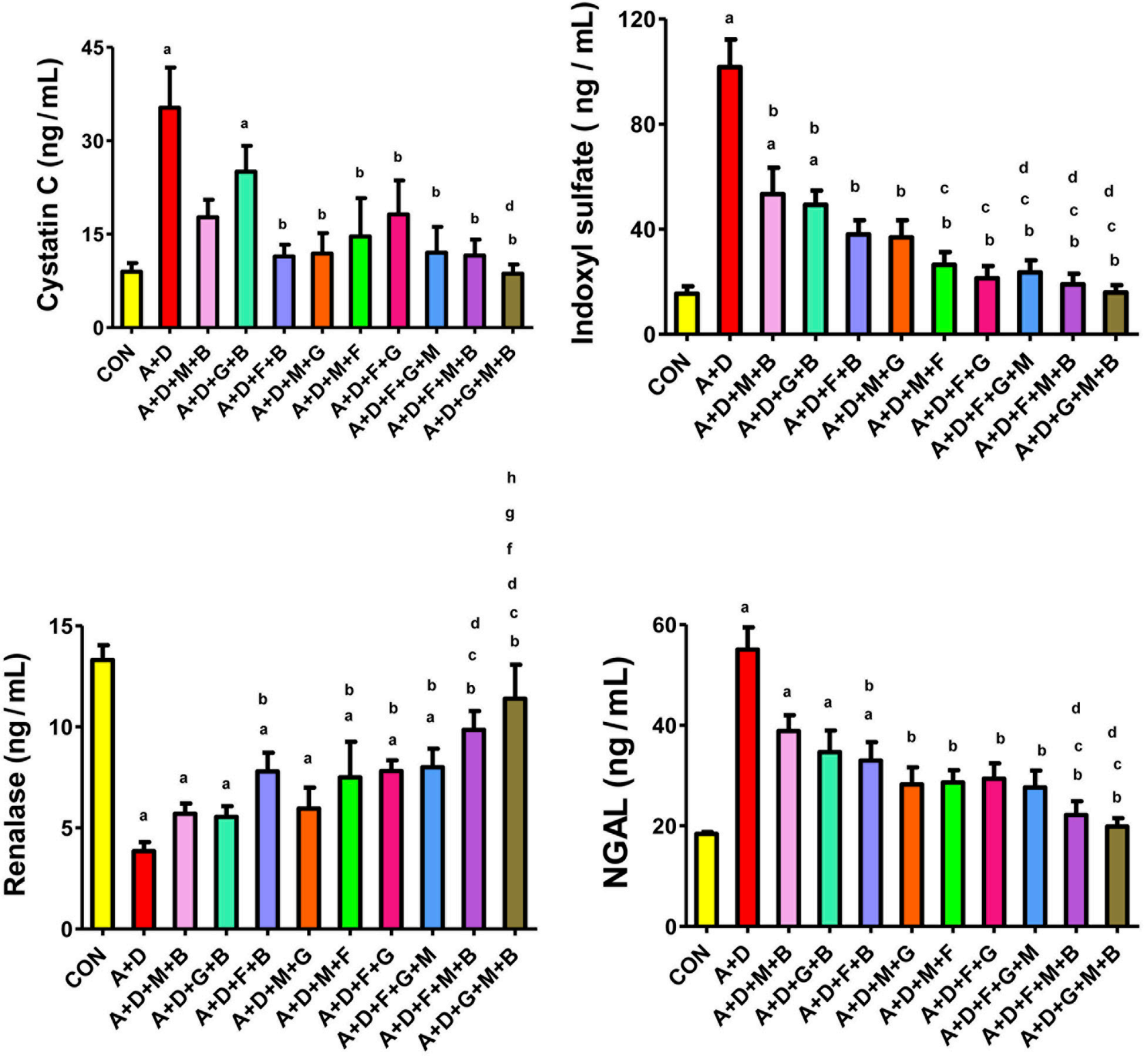
Effect of different combinations of flaxseed (F, 15%^w/w^), betaine (B, 200 mg/kg), melatonin (M, 10 mg/kg), and gum acacia (G, 15%^w/v^) on the plasma activity of cystatin C, concentrations of neutrophil gelatinase-associated lipocalin (NGAL) and indoxyl sulfate, and renalase activity in control (CON) rats and rats treated with both adenine (A, 0.25%) and diabetes (D, 55 mg/kg). Each vertical column with bar represents the mean ± SEM (n = 6). Differences between the groups were assessed by one-way analysis of variance (ANOVA) followed by Bonferroni’s multiple comparison test. Statistical significance was set at p < 0.05. Letters above the bars indicate statistically significant differences as follows: (a) control group vs. all other groups. (b) A + D (untreated) group vs. all other A + D treated groups. (c) A + D + M + B group vs. all other A + D treated groups. (d) A + D + G + B group vs. all other A + D treated groups. (e) A + D + F + B group vs. all other A + D treated groups. (f) A + D + M + G group vs. all other A + D treated groups. (g) A + D + M + F group vs. all other A + D treated groups. (h) A + D + F + G group vs. all other A + D treated groups. (i) A + D + F + G + M group vs. all other A + D treated groups. (j) A + D + F + M + B group vs. all other A + D treated groups.

Adenine and STZ induced a significant increase in NGAL activity and cystatin C and indoxyl sulfate concentration, and a significant reduction in the renalase activity (Figure 1). All the treatment groups significantly reduced indoxyl sulfate concentrations. In addition, all the treatment combinations, except melatonin–betaine and GA–betaine, significantly improved cystatin C and NGAL levels. Finally, all the treatment groups, except melatonin–betaine, GA–betaine, and melatonin–GA, significantly increased renalase levels. The GA–melatonin–betaine combination yielded the most notable improvement across all the parameters compared with the other treatment combinations.

### 3.5 Inflammatory indices

**FIGURE 2:**
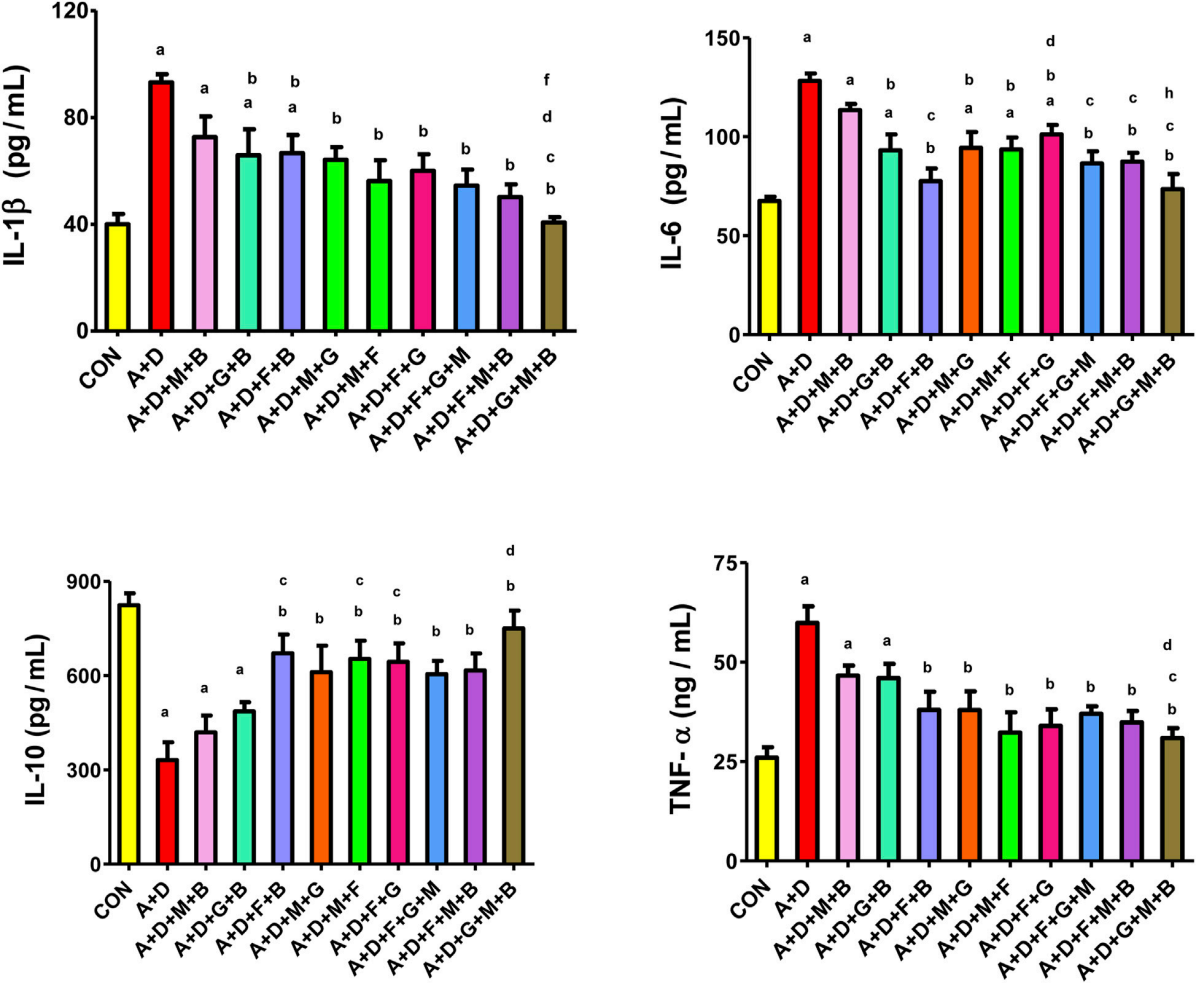
Effect of different combinations of flaxseed (F, 15%^w/w^), betaine (B, 200 mg/kg), melatonin (M, 10 mg/kg), and gum acacia (G, 15%^w/v^) on the on the plasma concentration of tumor necrosis factor (TNF-α), interleukin 1-beta (IL-1β), interleukin-6 (IL-6), and interleukin-6 (IL-10) in control (CON) rats and rats treated with both adenine (A, 0.25%) and diabetes (D, 55 mg/kg). Each vertical column with bar represents the mean ± SEM (n = 6). Differences between the groups were assessed by one-way analysis of variance (ANOVA) followed by Bonferroni’s multiple comparison test. Statistical significance was set at p < 0.05. Letters above the bars indicate statistically significant differences as follows: (a) control group vs. all other groups. (b) A + D (untreated) group vs. all other A + D treated groups. (c) A + D + M + B group vs. all other A + D treated groups. (d) A + D + G + B group vs. all other A + D treated groups. (e) A + D + F + B group vs. all other A + D treated groups. (f) A + D + M + G group vs. all other A + D treated groups. (g) A + D + M + F group vs. all other A + D treated groups. (h) A + D + F + G group vs. all other A + D treated groups. (i) A + D + F + G + M group vs. all other A + D treated groups. (j) A + D + F + M + B group vs. all other A + D treated groups.

Adenine and STZ induced a significant increase in IL-1β, IL-6, and TNF-α and significant suppression of IL-10 levels compared with the control group (Figure 2). All treatment combinations, expect melatonin–betaine, significantly reduced IL-1β and IL-6 levels. The GA–melatonin–betaine combination produced the most notable and significant mitigation in IL-1β compared with other treatment combinations. Furthermore, all treatment combinations, except melatonin–betaine and GA–betaine, significantly ameliorated the induced changes in IL-10 and TNF-α levels.

### 3.6 Oxidative stress indices

**FIGURE 3:**
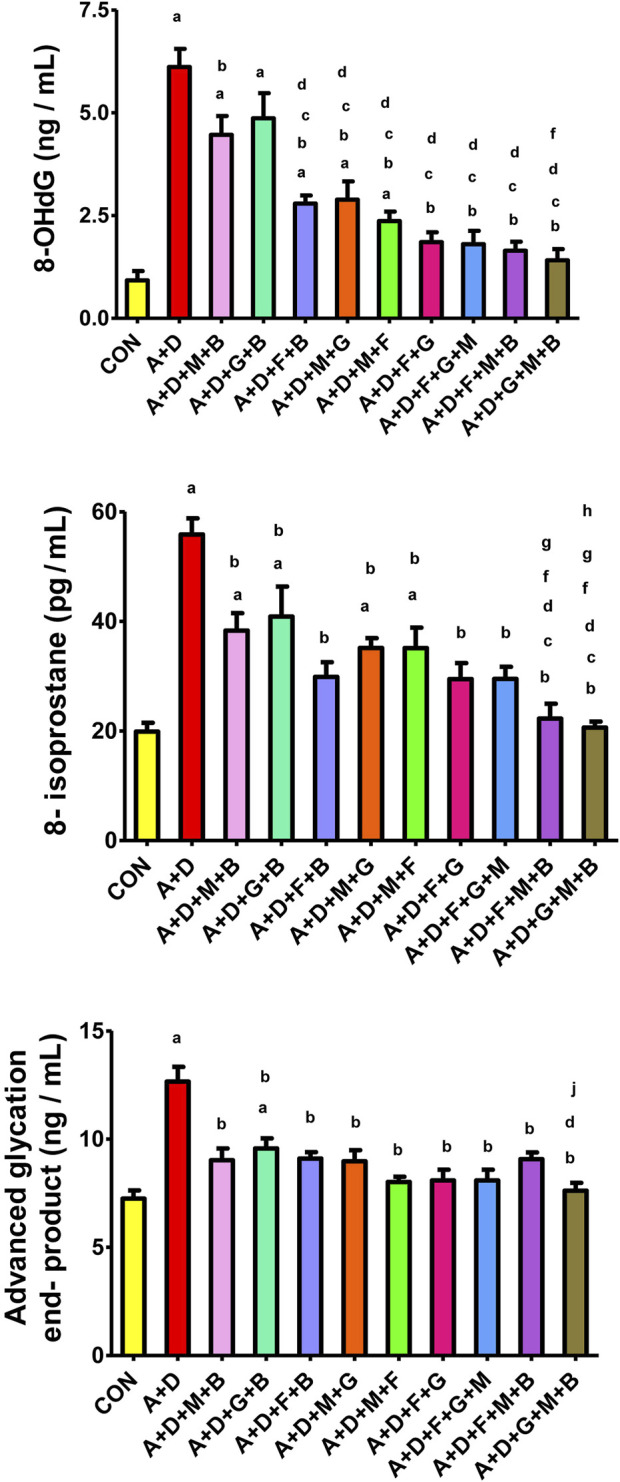
Effect of different combinations of flaxseed (F, 15%^w/w^), betaine (B, 200 mg/kg), melatonin (M, 10 mg/kg), and gum acacia (G, 15%^w/v^) on the on the plasma concentration of 8-isoprostane, advanced glycation end-products (AGEs), and 8-hydroxy-2′-deoxyguanosine (8-OHdG) in control (CON) rats and rats treated with both adenine (A, 0.25%) and diabetes (D, 55 mg/kg). Each vertical column with bar represents the mean ± SEM (n = 6). Differences between the groups were assessed by one-way analysis of variance (ANOVA) followed by Bonferroni’s multiple comparison test. Statistical significance was set at p < 0.05. Letters above the bars indicate statistically significant differences as follows: (a) control group vs. all other groups. (b) A + D (untreated) group vs. all other A + D treated groups. (c) A + D + M + B group vs. all other A + D treated groups. (d) A + D + G + B group vs. all other A + D treated groups. (e) A + D + F + B group vs. all other A + D treated groups.(f) A + D + M + G group vs. all other A + D treated groups. (g) A + D + M + F group vs. all other A + D treated groups. (h) A + D + F + G group vs. all other A + D treated groups. (i) A + D + F + G + M group vs. all other A + D treated groups. (j) A + D + F + M + B group vs. all other A + D treated groups.

Adenine and STZ induced a significant increase in 8-OHdG, 8-isoprostane, and AGEs (Figure 3), an effect that was significantly attenuated by all the treatment combinations, but it was more pronounced with the GA–melatonin–betaine combination.

**FIGURE 4:**
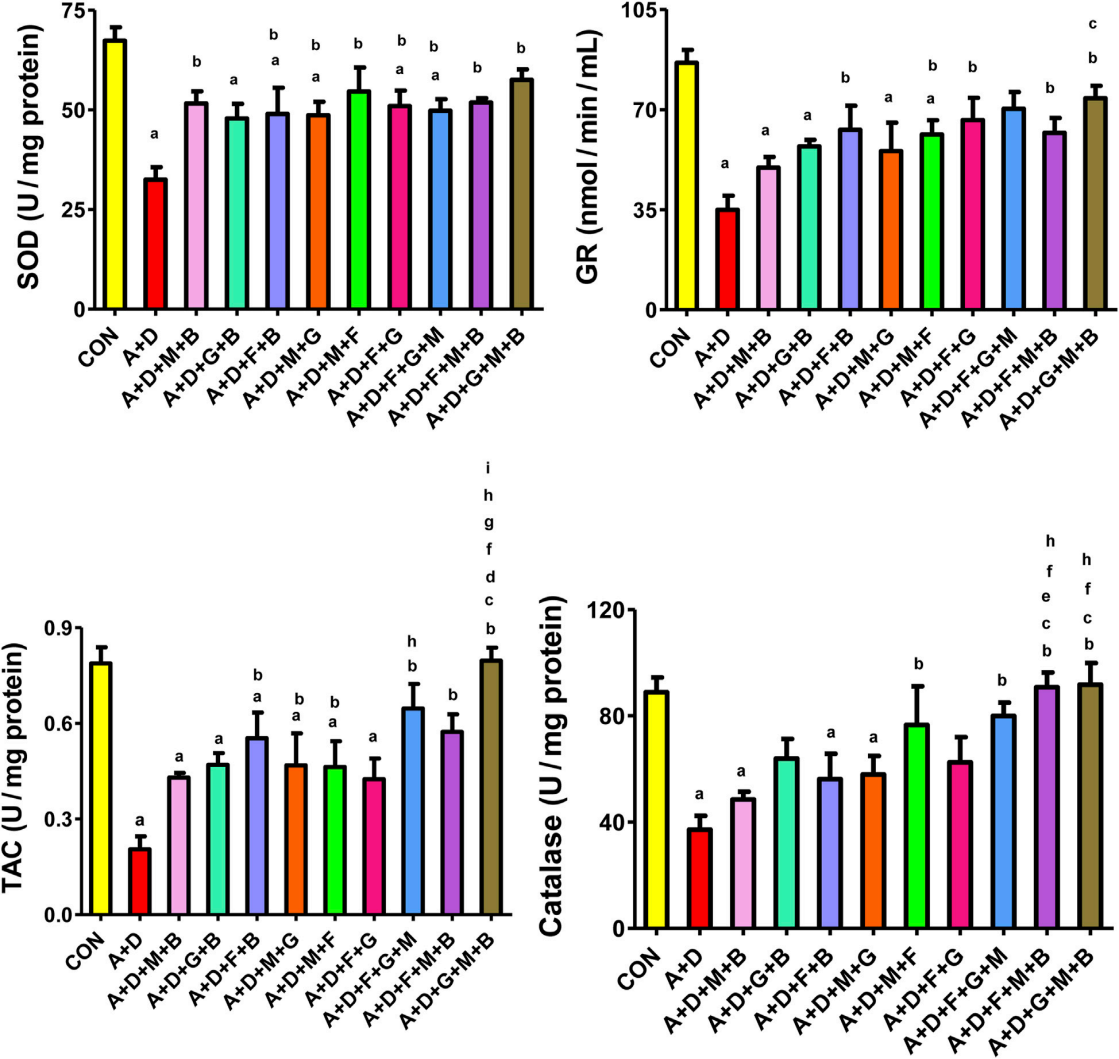
Effect of different combinations of flaxseed (F, 15%^w/w^), betaine (B, 200 mg/kg), melatonin (M, 10 mg/kg), and gum acacia (G, 15%^w/v^) on the plasma concentration of superoxide dismutase (SOD), catalase (CAT), total antioxidant capacity (TAC), and glutathione reductase (GR) in control (CON) rats and rats treated with both adenine (A, 0.25%) and diabetes (D, 55 mg/kg). Each vertical column with bar represents the mean ± SEM (n = 6). Differences between the groups were assessed by one-way analysis of variance (ANOVA) followed by Bonferroni’s multiple comparison test. Statistical significance was set at p < 0.05. Letters above the bars indicate statistically significant differences as follows: (a) control group vs. all other groups. (b) A + D (untreated) group vs. all other A + D treated groups. (c) A + D + M + B group vs. all other A + D treated groups. (d) A + D + G + B group vs. all other A + D treated groups. (e) A + D + F + B group vs. all other A + D treated groups. (f) A + D + M + G group vs. all other A + D treated groups. (g) A + D + M + F group vs. all other A + D treated groups. (h) A + D + F + G group vs. all other A + D treated groups. (i) A + D + F + G + M group vs. all other A + D treated groups. (j) A + D + F + M + B group vs. all other A + D treated groups.

Figure 4 presents the results of the different treatment groups on various oxidative stress indices. Adenine and STZ significantly decreased SOD, GR, TAC, and catalase activities compared with the control group. All treatment combinations, except GA–betaine, significantly attenuated the changes in SOD activity, with the effects generally comparable across groups. Treatment with flaxseed–betaine, melatonin–flaxseed, flaxseed–GA, flaxseed–melatonin–betaine, and GA–melatonin–betaine significantly increased GR activity. All the treatment groups except melatonin–betaine, GA–betaine, and flaxseed–GA significantly increased TAC activity, with the GA–melatonin–betaine combination producing the most marked and significant increase compared to the other combinations. Finally, treatment with melatonin–flaxseed, flaxseed–GA–melatonin, flaxseed–melatonin–betaine, and GA–melatonin–betaine significantly increased the catalase activity.

### 3.7 Histopathological changes

**FIGURE 5:**
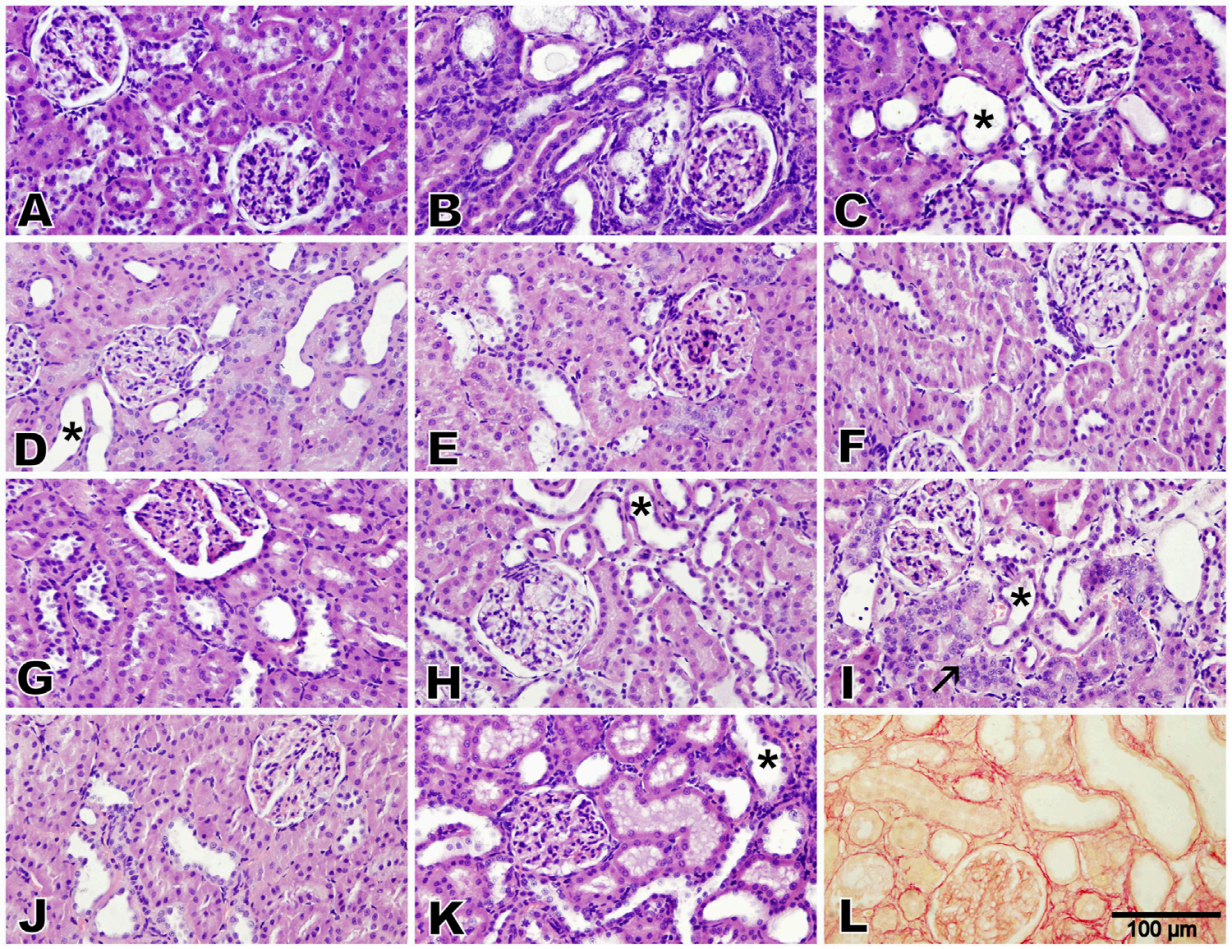
Photomicrographs of the renal cortex (Bar = 100 µm, H&E stain, except for L: Picrosirius red). The control group exhibited normal renal architecture with intact glomeruli and tubules (lesion score 0) **(A)**. Adenine and diabetes group exhibited severe cystic dilatation and pronounced basophilia of the renal tubules, numerous casts (lesion score 4), and marked peritubular fibrosis **(B,L)**. Groups treated with melatonin–betaine, flaxseed–GA, and flaxseed–GA–melatonin exhibited marked tubular dilatation and basophilia while maintaining intact glomeruli (lesion score 3), with the GA–melatonin-treated group showing notable tubular regeneration in the examined tissues **(C,H,I)**. Groups treated with GA–betaine, flaxseed–betaine, GA–melatonin, flaxseed–melatonin, flaxseed–melatonin–betaine, and GA–melatonin–betaine exhibited mild-to-moderate tubular dilatation while maintaining intact glomeruli (lesion score 2) **(D,E,F,G,J,K)**. The asterisks indicate cystic dilatation of renal tubules, and the arrow indicates regenerative renal tubules.

Microscopic analysis of the renal cortex of the rats in the control group exhibited a normal renal architecture, which was characterized by intact glomeruli and tubules (lesion score: 0) (Figure 5A). The adenine- and STZ-treated group displayed severe cystic dilatation and pronounced basophilia of the renal tubules, accompanied by numerous cellular casts (lesion score: 4) (Figure 5B). Kidney tissues from rats treated with melatonin–betaine, flaxseed–GA, flaxseed–GA–melatonin, and GA–melatonin–betaine showed significant tubular dilatation and basophilia while preserving intact glomeruli, earning a lesion score of 3 (Figures 5C,H,I). Notably, kidney tissues from rats treated with the flaxseed–GA–melatonin combination showed evidence of tubular regeneration in the examined tissues. Kidney tissues from rats treated with GA–betaine, flaxseed–betaine, melatonin–GA, melatonin–flaxseed, and flaxseed–melatonin–betaine demonstrated mild-to-moderate tubular dilatation while maintaining intact glomeruli, with each receiving a lesion score of 2 (Figures 5D–G,J). Additionally, marked peritubular fibrosis was noted in the kidney tissues of the groups treated with only adenine and STZ by Picrosirius red stain (Figure 5L).

## 4 Discussion

DM is a major health concern and a leading cause of CKD worldwide, resulting in substantial impairments in the quality of life, a higher incidence of premature mortality, increased risk of major cardiovascular events, and a significant burden on health-care systems (Francis et al., 2024). A recent international consensus emphasized the need for novel therapies to prevent the development and progression of kidney disease and to mitigate some of its associated health and socioeconomic burdens (Francis et al., 2024).

The pathological progression of kidney disease in DM is driven by a combination of metabolic, hemodynamic, inflammatory, and fibrotic factors involving multifaceted signaling pathways that collectively contribute to the gradual decline in the renal structure and function (Sinha and Nicholas, 2023). Effective treatment strategies should not only maintain glycemic control but also target and reverse the pathological mechanisms that drive disease progression. Several phytochemicals that target key pathological pathways in diabetes and CKD have been shown to reverse some of the underlying mechanisms implicated in DM-associated renal function decline (Al Za’abi et al., 2018; Al Za’abi et al., 2021a; Al Za’abi et al., 2021b). Consequently, these compounds may be of value as adjunct therapies alongside currently available medications, potentially improving patient outcomes.

In this study, we evaluated the effect of different combinations of flaxseed, GA, melatonin, and betaine in a rat model of experimentally induced diabetes and CKD. These natural products have previously been investigated for their potential to improve the glucose metabolism and/or renal function and are known to possess potent antioxidant and anti-inflammatory properties (Al Za’abi et al., 2018; Al Za’abi et al., 2021a; Al Za’abi et al., 2021b).

The induction of diabetes and CKD using STZ and adenine, respectively, in the experimental animals resulted in significant hyperglycemia, weight loss, polyuria, impaired renal function and structure, and increased oxidative stress accompanied by a compromised antioxidant defense system and dysregulated inflammatory markers. These features closely mirror the clinical and pathophysiological features of CKD in DM. Treatment with the two-drug and three-drug combinations was associated with the attenuation of most of these pathological changes to varying degrees. Notably, the GA–melatonin–betaine combination was linked to the most significant glycemic control and overall improvement across the majority of the measured indices, including urinary biochemical markers, systemic inflammation, oxidative stress, and renal function.

GA is a complex heteropolysaccharide chain rich in soluble fibers that has been widely investigated in different disease models. GA was found to improve several markers of renal function, oxidative stress, and inflammation, including serum urea, uric acid, creatinine, phosphorus, malondialdehyde, TAC, and C-reactive protein (Al-Jubori et al., 2023). Our findings align with previous reports and, for the first time, suggest that GA’s effects may be enhanced when combined with melatonin and betaine. Notably, this combination resulted in a more substantial restoration of most of the measured indices than other GA-containing combinations. Although the exact mechanism underlying this finding is unclear, it possibly involves a combination of pharmacokinetic and pharmacodynamic interactions. Recently, GA was shown to improve the bioavailability and absorption of omega-3 long-chain fatty acids in rats (Couëdelo et al., 2022), which could also extend to melatonin, given its high lipid solubility (Costa et al., 1995). Moreover, the physicochemical properties of GA are known to confer a pro-absorptive effect when administered concomitantly with various agents, as demonstrated with acetaminophen, polyethylene glycol 4000, and L-glucose (Codipilly and Wapnir, 2004). Further studies are required to clarify the specific pharmacokinetic and pharmacodynamic mechanisms underlying the observed effects.

GA is also recognized as an effective prebiotic agent that supports the growth of the gastrointestinal microbiome (Alobaidi, 2024). This has two implications in our present work: first, given that the microbiota directly influences drug absorption and metabolism (Zhao et al., 2023), it is plausible that GA favorably enhanced the metabolism and absorption of melatonin and betaine when added to the combination. Second, considering the pathological role of intestinal dysbiosis in the pathogenesis of CKD (Stavropoulou et al., 2021), it is possible that this prebiotic property of GA independently contributed to improving the renal outcomes by positively modulating the gut–kidney axis. In support of this, CKD progression was ameliorated and uremic toxins were blunted in experimental rats with gut microbiota modified using lactulose (Sueyoshi et al., 2019).

The observed improvements in the renal indices may partly be explained by the stronger glycemic control observed with the GA–melatonin–betaine combination, which may have contributed to the attenuation of some of the pathological processes driving renal function deterioration. GA is a viscous, water-soluble fiber that likely exerts hypoglycemic effects by reducing postprandial glucose absorption (Ibrahim et al., 2023). There is also evidence that GA regenerates β-cells and exerts insulin-like action by enhancing muscular and adipose tissue glucose uptake and inhibits hepatic gluconeogenesis (Ibrahim et al., 2023). Clinical trials have shown that GA improves blood glucose levels and reduces carbohydrate intake in patients with metabolic syndrome (Jarrar et al., 2021), further supporting the role of GA in blood glucose regulation.

In addition to these effects, GA possesses antioxidant and anti-inflammatory properties, as demonstrated in this work and elsewhere (Ibrahim et al., 2023; Shanab et al., 2023). The antioxidant capacity of GA is hypothesized to stem from its lysine, tyrosine, and histidine amino acid residues, which have strong antioxidant properties (Xu et al., 2017; Ibrahim et al., 2023), and its rich content of phenolic acids, which act as electron donors and serve as free radical scavengers (Shanab et al., 2023). The immunomodulatory mechanisms of GA are not fully established but are assumed to be driven by its derivative, butyrate, which regulates the expression of inflammatory mediators *via* the NFκB pathway (Al-Jubori et al., 2023).

Melatonin is an endogenous hormone produced by the pineal gland that regulates the circadian rhythm and is widely used as a supplement for managing insomnia (Delpino et al., 2021). It has been shown to regulate glucose homeostasis by stimulating glucose transport to skeletal muscle cells through the insulin receptor substrate-1/phosphoinositide 3 (IRS1/PI3)-kinase pathway (Zhu et al., 2023). Clinical trials have shown that melatonin supplementation can improve insulin sensitivity, fasting blood glucose, and HbA1c and glycemic variability (Delpino et al., 2021; Martorina and Tavares, 2023). In the current study, we demonstrate that the glucose-lowering effect of melatonin is significantly enhanced when administered in combination with GA and betaine compared to other combinations. This suggests that melatonin either has an independent effect or works synergistically with GA and betaine to improve glycemic control in the experimental animals. Melatonin receptors regulate insulin secretion from pancreatic β cells through three major intracellular signaling pathways: the adenylyl cyclase/cAMP pathway, the cGMP pathway, and the phospholipase C/inositol triphosphate pathway (Sharma et al., 2015). Recently, Patel et al. (2022) demonstrated that melatonin also stimulates β-cell regeneration and reduces apoptosis in STZ-induced diabetic mice.

The current findings further corroborate the well-established renoprotective role of melatonin across diverse experimental models of membranous nephropathy, lupus nephritis, hypertensive nephrosclerosis, acute ischemic kidney injury, unilateral ureteral obstruction-induced renal injury, treatment-induced nephrotoxicity, and contrast-induced AKI (Stacchiotti et al., 2002; Ozbek et al., 2009; Patschan et al., 2012; Wu et al., 2012; Cheng et al., 2014; dos Santos et al., 2018; Al Za’abi et al., 2021a). The renoprotective effects of melatonin have predominantly been attributed to its antioxidant, antiapoptotic, and anti-inflammatory properties. Melatonin is a potent antioxidant, with an indole ring and side chains that confer a broad-spectrum scavenging activity against a wide range of reactive oxygen and nitrogen species (Tan et al., 2015). Moreover, its metabolites retain free radical-neutralizing properties, thus amplifying melatonin’s overall antioxidant efficacy (Tan et al., 2015). Further evidence also suggests that melatonin regulates the gene expression of several antioxidant enzymes (Emamgholipour et al., 2016).

Furthermore, melatonin exerts significant anti-inflammatory actions through multiple mechanisms, including the inhibition of pro-inflammatory cytokines such as IL-1, IL-6, IL-8, and TNF; the downregulation of 5-lipoxygenase; and the reduction of leukocyte adhesion (Cho et al., 2021). These effects have been demonstrated in both animal studies and human clinical trials for different inflammatory conditions (Cho et al., 2021). In this work, melatonin administered in combination with GA and betaine was associated with the most pronounced anti-inflammatory effect compared to other combinations. Notably, the melatonin–betaine combination did not significantly attenuate any of the inflammatory markers, whereas other melatonin-containing combinations showed only moderate effects. This is in contrast to a previous study that demonstrated that melatonin–betaine exerted potent anti-inflammatory actions in rodents with cisplatin-induced nephrotoxicity (Al Za’abi et al., 2021a). In the context of diabetic kidney disease, melatonin and betaine appear to exhibit weak anti-inflammatory effects, whereas the inclusion of GA appears to significantly enhance the anti-inflammatory actions of this combination.

Betaine is a methyl derivative of glycine that is primarily obtained from dietary sources but is also endogenously synthesized in the liver from choline (Alvarenga et al., 2022). Betaine deficiency has been linked with diabetes, metabolic syndrome, dyslipidemia, and cardiovascular risk factors (Lever and Slow, 2010). On the other hand, betaine supplementation has been shown to reduce inflammatory markers and improve glycemic control, insulin resistance, renal function, liver injury, adipose dysfunction, and intestinal barrier integrity (Arumugam et al., 2021; Alvarenga et al., 2022).

Betaine is stored in the kidney and liver, and serves three important physiological functions: it acts as an osmolyte that regulates cell volume, it acts as a chemical chaperone that protects against protein degradation, and it serves as a methyl donor in the methionine cycle which detoxifies homocysteine to methionine—a process that is crucial for numerous cellular functions (Lever and Slow, 2010; Dobrijević et al., 2023). The osmoregulatory function of betaine assists in protecting renal cells from osmotic stress and electrolyte imbalances (Alvarenga et al., 2022; Dobrijević et al., 2023). In this study, the GA–melatonin–betaine combination normalized uric acid, urea, and phosphorus in STZ- and adenine-treated animals more prominently than the GA–melatonin combination. This enhanced effect may be partially attributed to the osmoregulatory properties of betaine. In addition, betaine has been shown to exert direct anti-hyperuricemic actions, where it modulates several renal uric acid and organic anion transporters (Liu et al., 2013).

Although homocysteine is commonly associated with cardiovascular diseases, there is increasing evidence supporting its strong correlation with CKD and renal injury (Shih et al., 2022; Chen et al., 2023). Proposed mechanisms include homocysteine’s cytotoxic effects, activation of profibrotic transcriptional factors, reduced glomerular filtration associated with hyperhomocysteinemia, and increased production of free radical species (Shih et al., 2022; Chen et al., 2023). Consequently, the homocysteine detoxification action of betaine may have contributed to the observed improvements in renal function with the GA–melatonin–betaine combination. The antioxidant and anti-inflammatory profile of this combination was also greater than that of the GA–melatonin combination alone. Although betaine itself is not a direct free radical scavenger, experimental *in vitro* evidence suggests that its antioxidant effects are mediated through the upregulation of endogenous non-enzymatic antioxidant defense systems and through the formation of physical protective barriers around cells (Zhang et al., 2016). Furthermore, betaine suppresses the nuclear factor-kappa B (NF-κB) signaling pathway, which regulates the expression of several pro-inflammatory effector molecules, including TNF-α and IL-1β (Zhao et al., 2018).

Flaxseed, which is rich in alpha-linoleic acids, fiber, flavonoids, and lignans, is a widely used dietary supplement (Mueed et al., 2022). Its therapeutic potential in attenuating diabetes-induced CKD in experimental rodents has been established previously (Al Za’abi et al., 2021b). This finding was corroborated in clinical trials, where flaxseed was found to improve fasting blood glucose, glycemic response, and various biomarkers of inflammation (Kavyani et al., 2023; Musazadeh et al., 2024). However, in the current study, flaxseed-containing combinations did not yield results comparable to the other combinations. One possibility is that the laxative properties of flaxseed may have compromised the absorption of the other components. Alternatively, the induction of cytochrome P450 enzymes by flaxseed lignans (Defries et al., 2021) may have reduced the bioavailability of the other components in the treatment combinations. Further investigation is required to clarify these potential interactions.

Taken together, our findings suggest that combining GA, melatonin, and betaine may offer a novel complementary therapeutic approach for attenuating the pathophysiological features of diabetes and CKD. However, the relevance of this finding to human therapy remains to be established. Although our findings suggest a potential synergistic effect, the underlying mechanisms were not explored in detail and require further mechanistic validation. Future studies should, therefore, aim to investigate the molecular pathways influenced by this combination, examine potential pharmacokinetic interactions, and evaluate long-term outcomes and clinical efficacy in human subjects.

In conclusion, the use of various combinations of GA, melatonin, betaine, and flaxseed attenuated, to different degrees, the effects of experimentally induced CKD and diabetes in rats treated with adenine and STZ. Among these combinations, the GA–melatonin–betaine combination produced the most significant reduction in blood glucose levels, accompanied by an overall improvement in renal function, renal injury markers, oxidative stress, and inflammation. We hypothesize that the observed improvement in CKD progression may be related to the stronger glycemic control achieved by this combination, coupled with complementary molecular, pharmacokinetic, and pharmacodynamic mechanisms. Our findings underscore the therapeutic potential of the GA–melatonin–betaine combination in offering multifaceted protection against diabetes and CKD. However, further research is needed to validate these findings, examine the exact mechanisms underlying these interactions, and explore the long-term clinical efficacy and safety of this combination for managing diabetic CKD in humans.

## Data Availability

The original contributions presented in the study are included in the article/supplementary material; further inquiries can be directed to the corresponding author.
